# Development and reproducibility of a computed tomography-based measurement of renal sinus fat

**DOI:** 10.1186/1471-2369-12-52

**Published:** 2011-10-04

**Authors:** Meredith C Foster, Shih-Jen Hwang, Stacy A Porter, Joseph M Massaro, Udo Hoffmann, Caroline S Fox

**Affiliations:** 1 National Heart, Lung, and Blood Institute's Framingham Heart Study, 73 Mt. Wayte Avenue, Suite 2, Framingham, Massachusetts, 01702 USA; 2 Department of Epidemiology, Harvard School of Public Health, Boston, Massachusetts, USA; 3 Center for Population Studies, National Heart, Lung, and Blood Institute, National Institutes of Health, Bethesda, Maryland, USA; 4 Harvard Medical School, 25 Shattuck Street, Boston, Massachusetts, 02115, USA; 5 Department of Biostatistics, Boston University School of Public Health, 715 Albany Street, T4E, Boston, Massachusetts, 02118 USA; 6 Department of Radiology, Massachusetts General Hospital, 55 Fruit Street, Boston, Massachusetts, 02114 USA; 7 Division of Endocrinology and Metabolism, Brigham and Women's Hospital, Harvard Medical School, Boston, Massachusetts, USA

## Abstract

**Background:**

Renal sinus fat may mediate obesity-related vascular disease, although this fat depot has not been assessed in a community-based sample. We sought to develop a protocol to quantify renal sinus fat accumulation using multi-detector computed tomography (MDCT).

**Methods:**

Protocol development was performed in participants in the Framingham Offspring cohort who underwent MDCT. Volumetric renal sinus fat was measured separately within the right and left kidneys, and renal sinus fat area within a single MDCT scan slice was measured in the right kidney. Due to the high correlation of volumetric and single-slice renal sinus fat in the right kidney (Pearson correlation [*r*] = 0.85, p < 0.0001), we optimized a single-slice protocol to capture renal sinus fat in the right kidney alone. Pearson correlation coefficients were used to compare to assess the correlation of volumetric and single-slice renal sinus fat in the right kidney with other measures of adiposity. Inter- and intra-reader reproducibility was assessed using intra-class correlation coefficients.

**Results:**

Single-slice measurements were obtained in 92 participants (mean age 60 years, 49% women, median renal sinus fat 0.43 cm^2^). Intra- and inter-reader intra-class correlation coefficients were 0.93 and 0.86, respectively. Single-slice renal sinus fat was correlated with body mass index (*r *= 0.35, p = 0.0006), waist circumference (*r *= 0.31, p = 0.003), and abdominal visceral fat (*r *= 0.48, p < 0.0001). Similar correlations were observed for volumetric renal sinus fat in the right kidney.

**Conclusions:**

Measuring renal sinus fat is feasible and reproducible using MDCT scans in a community-based sample.

## Background

Chronic kidney disease (CKD) is a major global health concern [1,2]. CKD affects over 13% of adults in the United States [3] and recent estimates in European adults indicate that stage 3 to 5 CKD affects about 4-7% of men and 6-10% of women [4]. CKD is associated with cardiovascular disease [5,6] and increased mortality [7,8] in addition to diabetes, hypertension, smoking, and obesity [9-16].

Obesity has reached epidemic proportions, with an estimated 1.3 billion adults worldwide either overweight or obese [17]. In the United States, the prevalence of obesity has continued to increase over the past 50 years [18], and the most recent reports indicate that one-third of American adults are obese, with an additional one-third overweight [19]. In 2002, the prevalence of obesity was greater than 20% in at least half of the countries in the European Union [20]. While obesity as defined based on body mass index (BMI) is associated with the development of CKD [12-16], reports indicate that measures of abdominal adiposity are also associated with CKD [21-24]. The observed associations of abdominal adiposity with CKD suggest that regional fat accumulation and ectopic fat, defined as the accumulation of fat within and around non-adipose tissues and organs [25], may mediate the relation of obesity to renal function. Within the kidney, ectopic fat can accumulate within the renal sinus, a renal cavity that also contains the renal artery, renal vein, lymphatic vessels, and nerves and has been observed in humans [26-28] and in an animal model of diet-induced obesity [29]. It is hypothesized to impair renal function through compression of renal structures, the release of locally acting molecules, or lipotoxicity in renal tissue.

Because the effects of such organ-specific fat accumulation are not captured using traditional anthropometric measurements of adiposity, radiographic techniques are necessary to investigate the potential role of kidney-specific fat accumulation in renal function. In the Framingham Heart Study, we have collected abdominal multi-detector computed tomography (MDCT) scans in a large sample of participants from the Offspring and Third Generation cohorts and have visualized the renal sinus. However, the feasibility and reproducibility of a MDCT-based renal sinus fat measurement has not been well-studied in a community-based sample. Thus, we sought to develop a method to measure renal sinus fat accumulation using MDCT in a sample from the Framingham Heart Study and to assess its reproducibility.

## Methods

### Study sample

The original Framingham Heart Study cohort was established in 1948 and consisted of 5209 adults between 28 and 62 years old from Framingham, Massachusetts. The Offspring cohort was established in 1971 and consisted of 5124 children and spouses of children of the original cohort. The Third Generation cohort was established in 2002 and consisted of 4095 participants with at least one parent in the Offspring cohort. The Framingham MDCT cohort consists of 3539 participants from the Offspring (*n *= 1422) and Third Generation (*n *= 2117) cohorts who had a MDCT scan between June 2002 and March 2005, as previously described [30]. A sample of 100 Offspring cohort participants from the MDCT cohort was used for the present analysis and was selected for equal representation of sex and 10-year age groups (35-44, 45-54, 55-64, 65-74, and 75-84 years old). Participants were randomly selected within strata defined by sex and age group. Participants provided written informed consent and this study was approved by the Boston University Medical Center and Massachusetts General Hospital institutional review boards and was conducted in accordance with the Helsinki Declaration.

### MDCT Scan Acquisition

Computed tomography images were captured using an 8-slice MDCT scanner (LightSpeed Ultra, General Electric; Milwaukee, WI, USA). The MDCT scan covered 125 mm in the abdomen with 25 5.0-mm slices above the S1 level (120 kVp, 400 mA, gantry rotation time 500 ms, table feed 3:1). Management and interpretation of MDCT scans was performed on a dedicated terminal using the Aquarius 3D Workstation (TeraRecon, Inc, San Mateo, CA, USA).

### Protocol Development

Renal sinus fat accumulation was measured using the Aquarius 3D Workstation body fat analysis template. Pixel density in Hounsfield units (HU) was used to identify adipose tissue based on a window width of -195 to -45 HU, centered on -120 HU.

The abdominal MDCT scans were originally collected to assess abdominal aortic calcification. Because of this, the entire kidneys were not visualized in our participants. Several characteristics suggested that enough of the right, but not the left kidney, was visible in our scans to measure renal sinus fat. We captured more of the right kidney than the left kidney on average in our study sample (14.6 versus 13.9 slices, respectively, p = 0.02). Additionally, we observed that volumetric renal sinus fat in the left kidney was correlated with the number of slices captured in the left kidney (Pearson correlation coefficient [*r*] = 0.29, p = 0.005) whereas volumetric renal sinus fat in the right kidney was not (*r *= 0.11, p = 0.27). This correlation in the left kidney was a concern, because it indicated that the measurement in the left kidney may be a function of the amount of kidney captured in the MDCT scans and may not reflect a true estimate of overall renal sinus fat in the left kidney. Based on these observations, we selected only the right kidney for further protocol development.

The next step in protocol development was to determine if a single slice that captures the renal sinus in the right kidney could represent volumetric renal sinus fat accumulation in the right kidney. In order to answer this question, we measured renal sinus fat area in a single slice from the right kidney and compared it to volumetric renal sinus fat measured using all visualized slices of the right kidney. The single-slice area measurement was correlated with the volumetric measurement in the right kidney (*r *= 0.85, p < 0.0001). Thus, we further optimized this single-slice protocol and developed a selection rule to identify the final renal sinus fat measurement slice.

**Figure 1:**
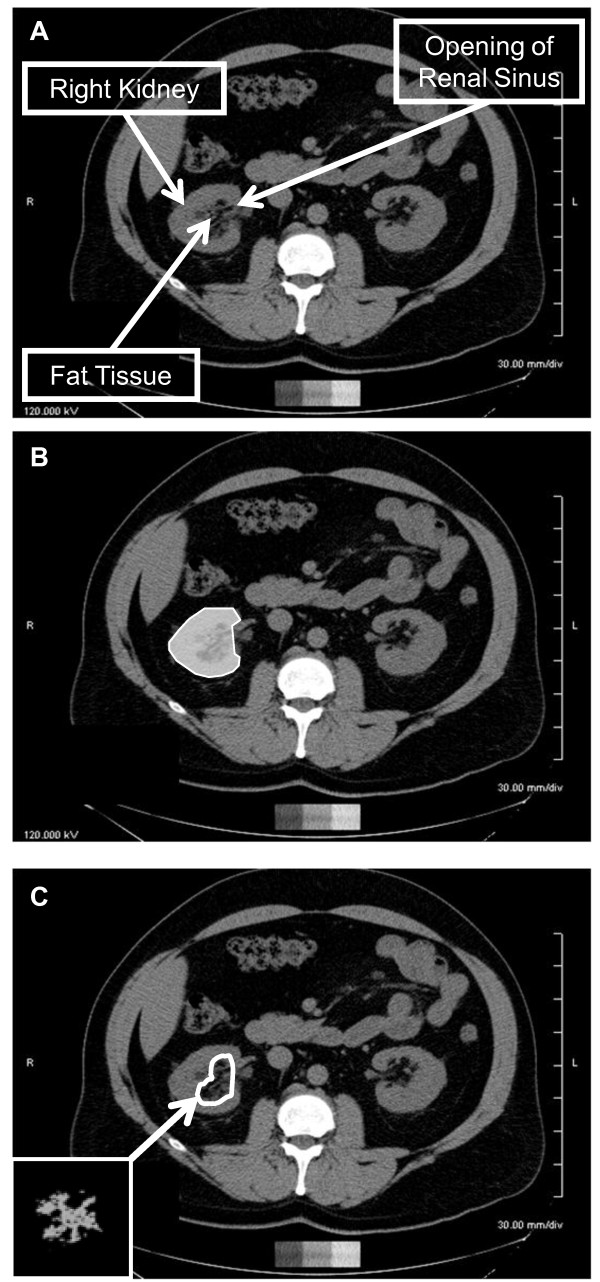
**Abdominal computed tomography scans demonstrating the renal sinus fat measurement technique**. A: Example of single abdominal computed tomography slice selected for renal sinus fat measurement. B: One measurement was taken in the right kidney. The reader manually traced within the border of the kidney. C: Visualization of renal sinus fat in slice measured in B using the Aquarius Workstation body fat analysis template (TeraRecon, Inc, San Mateo, CA, USA). The renal sinus is traced in this figure, highlighting the visualization of fat tissue observed in this region of interest.

Our final protocol consisted of a single-slice area measurement of renal sinus fat in the right kidney. To determine the slice with the maximum amount of fat within the renal sinus, the reader selected a range of several scan slices that appeared to have equivalent amounts of fat based on visual inspection. The single measurement slice was selected from this range using the following selection rule: if the range included an odd number of slices, then the middle slice of the range was traced; if the range included an even number of slices, then the anatomically more cranial of the two middle slices was traced. An example of a selected slice is presented in Figure 1a. The outer edge of the kidney in the selected slice was manually traced to separate it from the rest of the abdominal cavity (Figure 1b). The reader traced within the border of the kidney such that surrounding abdominal adipose tissue would be excluded from this measurement. The reader used a straight-line trace across the opening of the renal sinus from the dimples in the two adjacent lobes based on visual inspection (Figure 1b). Once traced, the Aquarius 3D Workstation software was used to measure the fat area within one slice of the right kidney (Figure 1c). Participants were excluded if (1) the renal sinus was not visualized or (2) if cysts or other structural abnormalities were present in the right kidney (*n *= 8).

### Covariate assessment

BMI (kg/m^2^) and waist circumference (cm) were determined based on anthropometric measurements taken by the trained clinic staff during the seventh Offspring examination cycle. During the clinic visit, weight was measured to the nearest pound, and height and waist circumference at the level of the umbilicus were measured to the nearest quarter-inch. Volumetric abdominal subcutaneous (SAT, cm^3^) and visceral adipose tissue (VAT, cm^3^) were assessed using the same set of abdominal MDCT scans, as previously described [31].

### Statistical methods

The final single-slice protocol was performed separately by two readers (MCF, SAP) for inter-observer variation and then repeated by the first reader (MCF) for intra-observer variation. Inter- and intra-reader reproducibility was assessed using intra-class correlation coefficients. Bland-Altman plots were utilized to assess potential systematic biases within the intra-reader and inter-reader repeated measurements. Correlations of single-slice renal sinus fat and volumetric renal sinus fat in the right kidney with BMI, waist circumference, SAT, and VAT were assessed using age- and sex-adjusted partial Pearson correlation coefficients. Statistical analyses were performed using SAS Version 9.2 (SAS Institute Inc; Cary, NC, USA).

## Results

### Sample characteristics

Among the 100 individuals in the reproducibility sample, 92 participants had abdominal MDCT scans that could be used for the single-slice measurement protocol. Characteristics of the reproducibility sample with renal sinus fat measurements are presented in Table 1. The mean age was 60 years and 52% (n = 48) were women. The mean BMI, waist circumference, and abdominal VAT volume were 27.7 kg/m^2^, 98.7 cm, and 2007 cm^3^, respectively.

**Table 1:** Demographic characteristics of the reproducibility sample at the 7^th ^Offspring Examination

Variable	N	Mean	Standard Deviation	Minimum	**25**^**th**^**percentile**	Median	**75**^**th**^**percentile**	Maximum
**Age (years)**	92	60	13	37	48	60	72	83

**Weight (kg)**	92	78.8	16.0	48.5	67.1	79.4	90.5	137.0

**Height (cm)**	92	168.4	9.0	150.5	160.0	168.0	175.6	190.5

**Body mass index (kg/m**^**2**^**)**	92	27.7	4.6	18.1	24.6	27.3	30.0	41.5

**Waist circumference (cm)**	92	98.7	12.7	71.8	89.9	99.1	106.4	132.1

**Subcutaneous Adipose Tissue (cm**^**3**^**)**	92	2930	1291	501	1971	2743	3410	6695

**Visceral Adipose Tissue (cm**^**3**^**)**	92	2007	1025	289	1249	1882	2758	4371

**Renal fat measures**								

**Right kidney, volumetric (cm**^**3**^**)**	91	5.62	5.40	0.0095	1.83	3.99	7.49	25.61

**Right kidney, one-slice (cm**^**2**^**)**	92	0.69	0.72	< 0.0048	0.17	0.43	1.07	2.95

### Distribution of renal sinus fat

Among participants with a volumetric renal sinus fat measurement in the right kidney (n = 91), the mean total fat volume was 5.62 cm^3^, ranging from 0.01 cm^3 ^to 25.61 cm^3^. No participants had an undetectable volumetric renal sinus fat measurement in the right kidney; the volumetric renal sinus fat measurement was missing for one participant. Among participants with an interpretable MDCT scan for the single-slice renal sinus fat measurement (n = 92), the mean renal sinus fat area was 0.69 cm^2^, ranging from undetectable (n = 3) to 2.95 cm^2^.

### Intra- and inter-reader reproducibility

**Figure 2:**
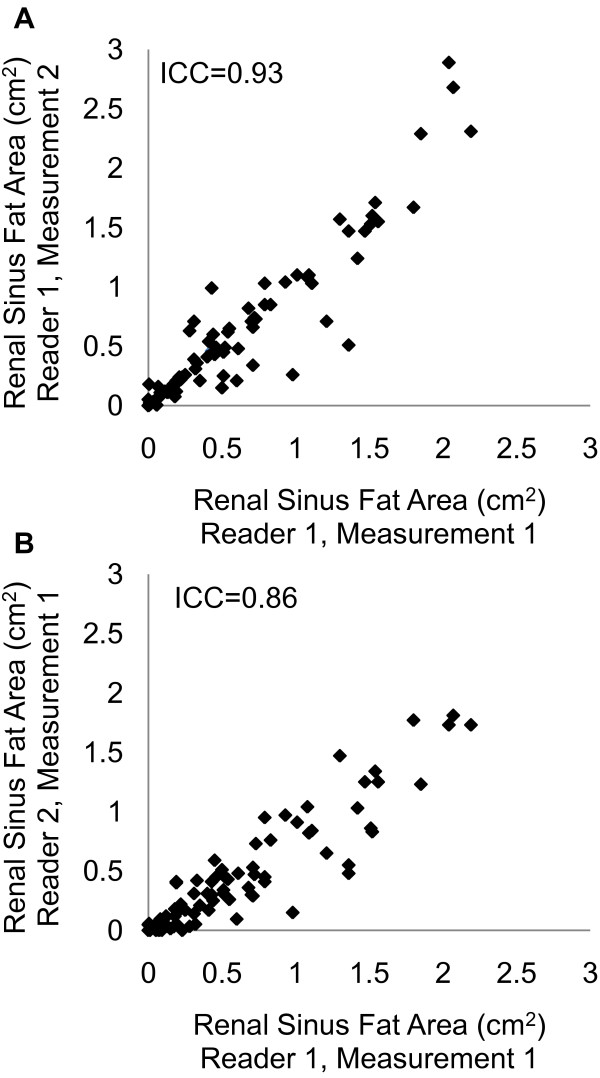
**Intra-reader and inter-reader single-slice renal sinus fat measurements**. Renal sinus fat measures between one reader (A) and two readers (B) are plotted. Intra-reader intra-class correlation coefficient (ICC) = 0.93 and inter-reader ICC = 0.86.

Intra- and inter-reader renal fat measurements are plotted in Figures 2a and 2b, respectively. The intra-reader intra-class correlation coefficient was 0.93 and the inter-reader intra-class correlation coefficient was 0.86 for our single-slice measurement protocol.

**Figure 3:**
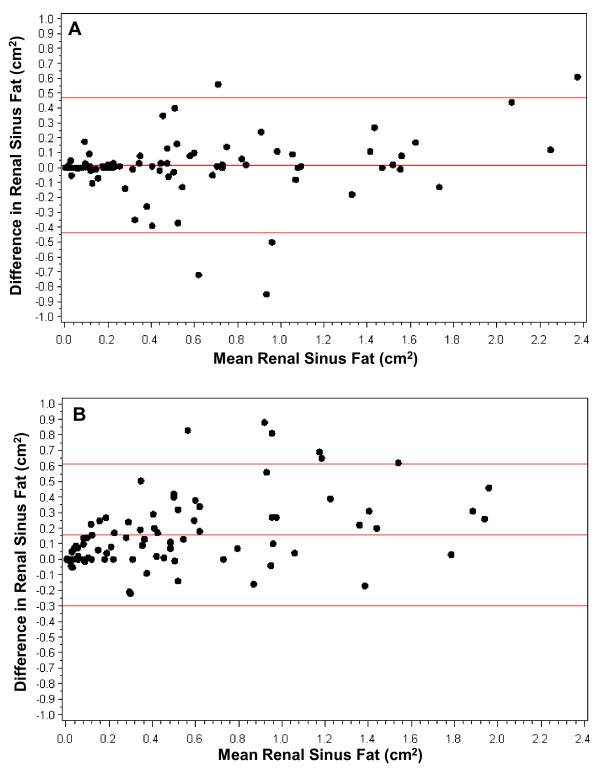
**Bland-Altman plots of (A) the intra-reader renal sinus fat measurements and (B) the inter-reader renal sinus fat area measurements**. The average of the repeated measurements is presented on the X-axis and the difference between the two measurements is presented on the Y-axis. The middle red line represents the mean difference between the repeated measures and the upper and lower red lines represent upper and lower confidence limits for the mean difference, respectively.

The Bland-Altman plot for the intra-reader repeated measurements is presented in Figure 3A. Overall, the mean difference between the repeated measurements was 0.018 cm^2 ^with upper and lower confidence limits of 0.435 cm^2 ^and -0.470 cm^2^, respectively, suggesting minimal systematic bias between the intra-reader measurements. The Bland-Altman plot for the inter-reader repeated measurements is presented in Figure 3B. Here, the mean difference between the repeated measures was 0.157 cm^2^, with upper and lower confidence limits of 0.299 cm^2 ^and -0.613 cm^2^, indicating that the first reader's measurements are on average slightly higher than the second reader's measurements. While this suggests a systematic bias in the inter-reader measurements, our main objective was to develop a single-slice protocol to use as a marker of overall renal sinus fat accumulation. This bias would likely not impact the ranking of individuals by renal sinus fat accumulation within each set of reader measurements.

### Correlation of renal sinus fat with adiposity measures

Age- and sex-adjusted partial Pearson correlation coefficients for single-slice renal sinus fat and volumetric renal sinus fat in the right kidney with measures of adiposity are presented in Table 2. Single-slice renal sinus fat was correlated with BMI (*r *= 0.35), waist circumference (*r *= 0.31), and VAT (*r *= 0.48, all *p *≤ 0.003). Similar correlations were observed for volumetric renal sinus fat.

**Table 2:** Age- and sex-adjusted partial Pearson correlation coefficients of renal sinus fat with adiposity measures in the reproducibility sample.

	Volumetric Renal Sinus Fat (n = 91)	Single-Slice Renal Sinus Fat (n = 92)
**Body Mass Index**	0.35 *p *= 0.0008	0.35 *p *= 0.0006

**Waist Circumference**	0.32 *p *= 0.002	0.31 *p *= 0.003

**Subcutaneous Adipose Tissue**	0.17 *p *= 0.12	0.17 *p *= 0.11

**Visceral Adipose Tissue**	0.49 *p *< 0.0001	0.48 *p *< 0.0001

## Discussion

Using a sample from the Framingham MDCT study cohort, we have shown that measuring renal sinus fat using computed tomography is feasible and reproducible in a community-based sample. During protocol development, we observed that a single-slice renal sinus fat measurement is highly correlated with volumetric renal sinus fat accumulation in the right kidney. We also observed that the single-slice and volumetric renal sinus fat measurements are similarly correlated with other measures of adiposity, including BMI, waist circumference, and abdominal VAT, suggesting that only one slice needs to be manually traced by the reader to assess renal sinus fat.

Computed tomography, among other imaging techniques, has been used to assess the development of renal lesions in humans, including renal sinus fat accumulation [32,33]. Previous small imaging studies using magnetic resonance imaging have demonstrated fat accumulation in the renal sinus of children with no known renal disease (n = 15 of 58 participants) [26] and adults [27]. Magnetic resonance imaging has also been used to assess renal sinus fat in 205 participants in the Pulmonary Edema and Stiffness of the Vascular System study, which consists of middle-aged and elderly participants with cardiovascular risk factors [28]. In the animal literature, renal sinus fat accumulation has been described in a rabbit model of diet-induced obesity [25,29]. In obese rabbits fed a high fat *ad lib *diet for 8-12 weeks, renal sinus mass was 61% greater than observed in lean control rabbits, with the majority of this increase due to a 2.6-fold increase in renal sinus fat accumulation [29]. It is proposed that such increases in renal sinus fat can compress renal structures, including the renal medulla, renal vein and lymph vessels, leading to changes in renal interstitial pressure [25]. Renal sinus fat may also lead to renal tissue damage through paracrine effects of locally released adipocytokines or lipotoxicity.

Lipid accumulation within renal tissues has also been observed in humans [34,35] and animal models [36-40]. The results from animal models support mechanisms of obesity-induced hypertension and renal dysfunction through oxidative stress, inflammation, and fibrosis within glomeruli and proximal tubules [41,42]. While renal lipid accumulation is important when considering the impact of obesity on renal function, this accumulation within renal cells is not captured in our measurement of renal sinus fat. Other techniques, including magnetic resonance imaging [43], are required to address renal intra-cellular lipid accumulation and its potential role in obesity-related renal dysfunction in humans.

The present study adds to the literature with the development of a protocol to quantify the accumulation of renal sinus fat in a community-based sample based on imaging techniques. Our study demonstrates that computed tomography can be used to develop a reproducible measurement protocol for renal sinus fat accumulation. Given the current obesity epidemic, ectopic fat depots may play an important role in organ dysfunction. This technique will allow for the characterization of the distribution of renal sinus fat and to assess its correlation with other clinical characteristics, which may lead to a better understanding of the role of adiposity in renal dysfunction and obesity-related vascular disease.

A major strength of our study is the use of a well-characterized, community-based sample that was not selected for obesity-related outcomes. There are limitations of our study that warrant mention. No *in vivo *gold standard measurement for renal sinus fat exists in human studies, which limits our ability to assess measurement validity. Our reproducibility sample is comprised of white participants from the Framingham Heart Study, which may limit the generalizability of our findings to other cohorts or ethnicities.

## Conclusions

In conclusion, renal sinus fat accumulation can be reproducibly measured using computed tomography in a sample from the Framingham MDCT cohort. In our protocol development, we observed that a single-slice measurement of renal sinus fat accumulation is correlated with volumetric renal sinus fat accumulation in the right kidney.

## Competing interests

The authors declare that they have no competing interests.

## Authors' contributions

MCF contributed to the conception and design of this study, analysis and interpretation of data, and drafting the manuscript. SJH contributed to the analysis and interpretation of data and revising the article critically for important intellectual content. SAP participated in data acquisition and revising the manuscript critically for important intellectual content. JMM and UH revising the manuscript critically for important intellectual content. CSF contributed to the conception and design of this study, analysis and interpretation of data, and drafting the manuscript and revising the manuscript for important intellectual content. All authors read and approved the final manuscript.

## Pre-publication history

The pre-publication history for this paper can be accessed here:

http://www.biomedcentral.com/1471-2369/12/52/prepub
